# Comparison of gait characteristics between affected and unaffected sides in patients with meniscus lesions: insights from dynamic analysis

**DOI:** 10.3389/fmed.2025.1683174

**Published:** 2025-10-27

**Authors:** Lin Ma, LiuFeng Xiao, Pan Huang, Dianwei Li, Yunjiao Wang, Peiyao Liang, Yonghua Chen, Lin Guo

**Affiliations:** Department of Sports Medicine, Southwest Hospital, Army Medical University, Chongqing, China

**Keywords:** gait analysis, meniscus, knee, walking, foot–ground kinematics

## Abstract

**Purpose:**

This study aims to elucidate the alterations in gait patterns following knee injuries by comparing the gait analysis of the affected and unaffected sides in patients with meniscus lesions. The findings may provide a valuable reference for the early detection of meniscus injuries.

**Methods:**

The study involved 37 patients diagnosed with meniscus lesions at the Sports Medicine Center of our hospital between December 2023 and November 2024. These patients were confirmed to have meniscus lesions through MRI and arthroscopic surgery. We employed the Dynamic Gait & Posture Analysis System (Shenzhen Xingzheng Technology Co., Ltd.) to analyze three-dimensional gait characteristics, comparing the differences in 3D gait between the affected and unaffected sides.

**Results:**

A total of 37 patients were included in the study, with an average age of 39.24 ± 14.06 years, comprising 18 males and 19 females. Among these patients, 18 had right-sided involvement and 19 had left-sided involvement, with an average disease duration of 20.54 ± 28.59 months. The results of gait phase analysis indicated that the affected side exhibited a shorter duration during the propulsion phase compared to the unaffected side (*p* < 0.05). Additionally, there were no significant differences in step velocity, step cadence, step length, toe-out angle, and stability support during single-leg stance between the affected and unaffected sides. Analysis of sagittal plane angles between the plantar surface and the ground revealed that the velocity of impact, the maximum dorsiflexion velocity at the beginning of the swing (MDVBW), and the maximum swing velocity on the affected side were all significantly lower than those on the unaffected side (*p* < 0.05). In contrast, the foot landing angle and angle of propulsion did not show significant differences between the sides. Furthermore, no significant differences were found in the coronal plane angles between the foot sole and the ground measurements, nor in the measurements of foot long axis alignment with the forward direction.

**Conclusion:**

Among patients with meniscal lesions, the affected limb showed reduced propulsion-phase proportion, impact velocity, MDVBW, and maximum swing velocity. These side-to-side differences likely reflect sagittal-plane pain-avoidance adaptations.

## Introduction

A torn meniscus of the knee is a prevalent sports injury, particularly associated with activities such as running, jumping, and high-intensity, prolonged exercise (1). The meniscus is a crescent-shaped fibrocartilage positioned between the femur and tibia that serves to enlarge the contact surface of the knee, maintain joint stability, transmit and disperse loads, and protect the articular cartilage (2). Meniscus tears can be acute, often caused by twisting injuries, or chronic due to overuse. They typically cause pain, swelling, restricted motion, and altered gait from pain avoidance, which reduces knee stability and increases joint stress. These changes accelerate cartilage damage, underscoring the importance of early functional detection.

Although imaging techniques are essential for diagnosing meniscal lesions, they primarily capture structural changes and provide limited insight into the early functional adaptations of the joint. In contrast, gait analysis offers a dynamic, noninvasive approach to evaluate joint mechanics by quantifying parameters such as stride length, cadence, swing phase, and support phase (3). Alterations in these variables reflect the biomechanical consequences of injury and may reveal abnormal loading or compensatory strategies that are not detectable on imaging alone.

Prior studies have reported gait changes after meniscectomy and in patients with lateral discoid meniscus lesions (4, 5). However, most emphasize between-group comparisons of variables such as knee moments or peak knee flexion angles, with relatively less attention to within-subject side-to-side differences and to less commonly reported propulsion-related metrics. Because pain-avoidance behaviors are often asymmetric, a within-patient comparison of the affected and contralateral limbs—particularly during the propulsion phase and in swing-phase velocity—may be more sensitive to clinically relevant dysfunction than group-level averages alone. Establishing robust, side-specific functional markers would complement imaging and provide actionable targets for rehabilitation.

The present study employed a multi-scenario stealth gait analysis system to compare sagittal and coronal gait parameters between the affected and unaffected sides in patients with meniscus lesions. By identifying specific gait asymmetries associated with meniscal injury, this research seeks to provide a more comprehensive understanding of its functional impact. These findings may serve as a foundation for early detection and individualized rehabilitation strategies, offering practical value for clinicians in optimizing treatment timing, preventing secondary joint damage, and improving long-term outcomes. We hypothesize that, relative to the contralateral limb, meniscal lesions lead to reduced propulsion-phase duration and swing velocity in the affected limb due to pain-avoidance strategies.

## Methods

### Participants

This study collected clinical data from patients diagnosed with knee meniscal injuries at our institution between December 2023 and November 2024. The research protocol was approved by the Ethics Committee (Approval No: KY202246).

#### Inclusion criteria

(1) Diagnosis of a grade III knee meniscus tear confirmed by MRI and arthroscopic surgery; (2) diagnosis established by a senior orthopedic surgeon with more than 10 years of clinical experience; (3) patients aged 18–60 years; (4) no previous history of knee trauma to avoid confounding pain-related effects on gait analysis.

#### Exclusion criteria

(1) Patients with other diseases affecting the lower limb musculoskeletal system; (2) patients who have previously undergone surgery on their lower limbs; (3) patients with abnormal lower limb alignment; (4) patients exhibiting abnormal nerve and muscle function in their lower limbs; (5) patients with foot deformities.

### Evaluation method

The Dynamic Gait & Posture Analysis System (Shenzhen Xingzheng Technology Co., Ltd.) was utilized in this study. The effectiveness of this system was evaluated by the China National Robot Quality Supervision and Inspection Center (Chongqing). According to the inspection report, across the eight selected gait cycle parameters, the average difference between this system and the VICON 3D infrared motion capture system (Vantage V5, Oxford Metrics) was 0.91%. The inspection report has been provided as an attachment for reference.

### Data collection

For each participant, a suitable detection insole was selected and the data acquisition module securely installed. The examiner instructed participants to place the insole in flat shoes before beginning the procedure. Prior to formal testing, each subject completed a three-minute familiarization walk at a self-selected pace along a 20-m walkway to ensure a stable and natural gait pattern.

The experimental protocol then required three 1-min walking trials on the same walkway at a comfortable, self-selected pace, guided by prompts displayed on the mobile control interface. During each trial, the gait analysis module continuously recorded real-time biomechanical signals and displayed the corresponding foot posture. Data was automatically transmitted to the back-end algorithm layer for subsequent processing and evaluation. Following each trial, data integrity was checked, and the average of the three valid trials was used for analysis.

Trials were excluded under the following conditions: the participant failed to complete the one-minute walk at a consistent pace (e.g., hesitation, abrupt acceleration, or stopping); the insole shifted within the shoe, resulting in unstable signal acquisition; sensor malfunction, missing data, or transmission errors occurred; or external disturbances such as obstacles, interruptions, or uneven surfaces interfered with gait.

### Evaluation indicators

Gait phase analysis included the proportions of the affected and unaffected sides during the stance, neutral, propulsion, and swing phases. The stance phase (first contact to foot flat) begins when the foot touches the ground; the neutral phase (mid- to late stance) continues until heel-off; the propulsion phase (heel-off to toe-off) ends when the forefoot leaves the ground; and the swing phase lasts until the next contact. Gait parameters in this study included:

(1) General parameters: gait speed (m/s), step frequency (steps/min), stride length (m), turning angle (°), and single-leg support stability.
(2) Sagittal plane angles: foot landing angle (°), propulsion angle (°), impact velocity (°/s), MDVBW (°/s); reflecting the peak dorsiflexion velocity of the ankle dorsiflexors, mainly the tibialis anterior, and maximum swing velocity (m/s).
(3) Foot–ground angles: pronation angle at touchdown (°), overall pronation angle (°), pronation velocity (°/s), lift height (cm), and the angle between the plantar surface and the ground at swing initiation (APSGBS, °; indicating foot orientation for effective toe clearance).
(4) Foot axis angles: swing width (cm) and foot deviation angle (°).

### Sample size

As within-subject comparisons are uncommon in previous studies, we estimated sample size based on step length data from a meniscal injury group and a healthy control group (5). The mean step length was 0.62 ± 0.06 m in the injury group and 0.68 ± 0.04 m in controls. Using an independent samples *t*-test with a two-sided *α* = 0.05 and 80% power, approximately 12 participants per group were required. To account for potential dropouts, 14–15 participants per group will be recruited. The effect size (Cohen’s *d*) was 1.18, indicating a large difference between groups. A total of 37 participants were enrolled in this study, meeting the minimum sample size requirement.

### Statistical analysis

All analyses were performed in SPSS (v26.0; IBM, Armonk, NY, USA). Normality of each variable was assessed with the Shapiro–Wilk test. Variables with normal distributions are reported as mean ± standard deviation and were compared using the paired-samples *t*-test; effect sizes are presented as Cohen’s *d* (for paired designs). Non-normal variables are reported as median (interquartile range) and were compared using the Wilcoxon signed-rank test; effect sizes are reported as *r* (computed as Z/ $\sqrt{N}$ ). Unless otherwise stated, tests were two-tailed, and statistical significance was set at *p* < 0.05.

## Results

### Demographic data

**Figure 1:**
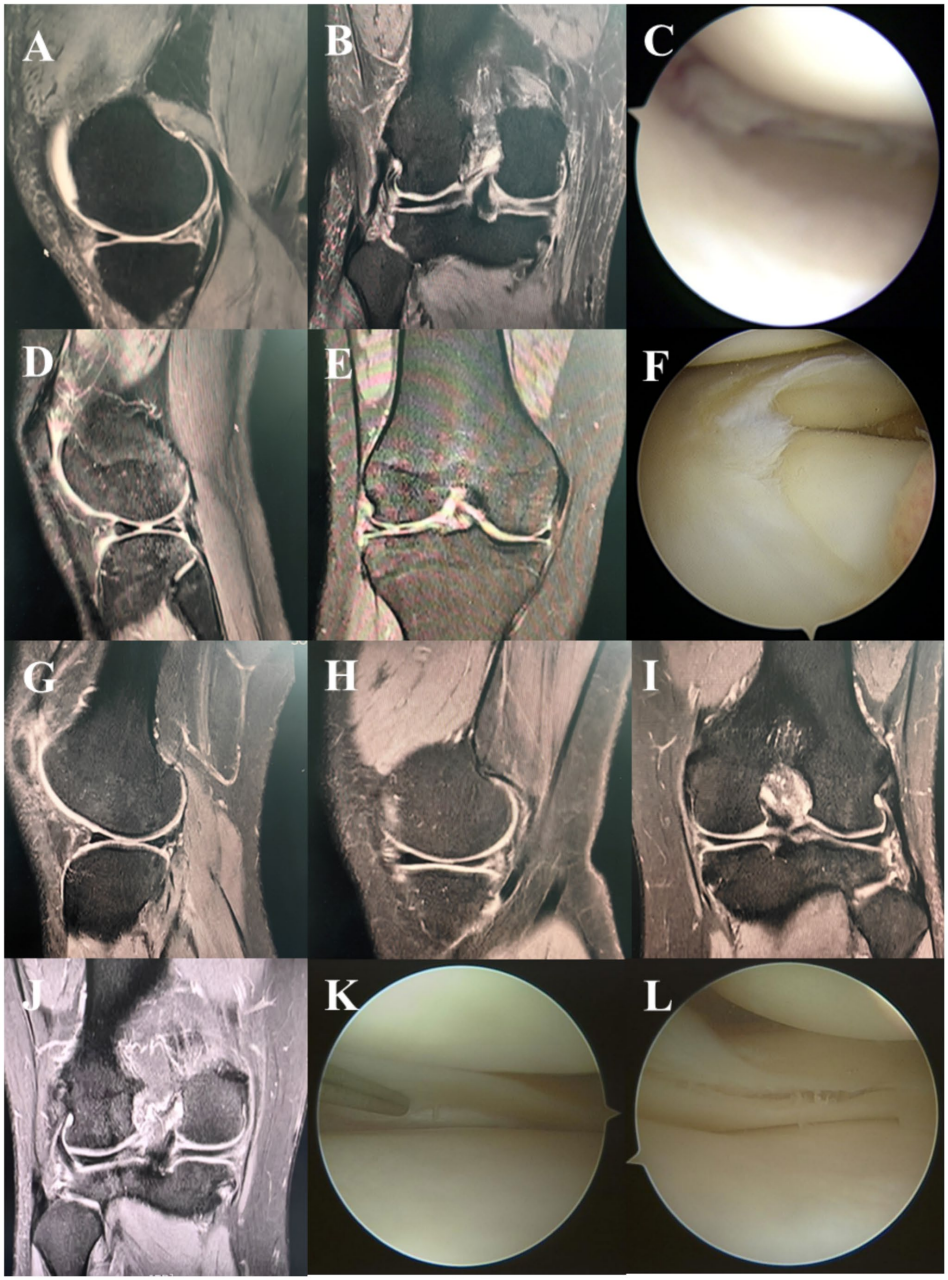
Typical cases. MRI sagittal **(A)**, coronal **(B)** and arthroscopic **(C)** manifestations of lateral meniscus injury. MRI sagittal **(D)**, coronal **(E)** and arthroscopic **(F)** manifestations of medial meniscus injury. MRI sagittal **(G,H)**, coronal **(I,J)** and arthroscopic **(K,L)** manifestations of both lateral and medial meniscus injuries.

A total of 37 patients were included in the study, with an average age of 39.24 ± 14.06 years, comprising 18 males and 19 females. Among these patients, 18 presented with right knee injuries, while 19 had left knee injuries, with an average disease duration of 20.54 ± 28.59 months (Table 1). All patients were diagnosed with meniscus injuries through MRI and confirmed via intraoperative arthroscopy (Table 1 and Figure 1).

**Table 1:** Characteristics of the participants.

Demographic	
Characteristic	*N* = 37
Age (yr)	39.24 ± 14.06 (37)
Range (minimum–maximum)	18–60
Gender	
Female, *n* (%)	19 (51.35%)
Male, *n* (%)	18 (48.65%)
BMI	23.69 ± 3.82 (22.68)
Range (minimum–maximum)	18.90–37.45
Affected side	
Right, *n* (%)	18 (48.65%)
Left, *n* (%)	19 (51.35%)
Symptom duration (months)	9 (2 ~ 31.5)
Range (minimum–maximum)	1–120
Types of meniscal tears	
Medial, *n* (%)	13 (35.14%)
Lateral, *n* (%)	19 (51.35%)
Both medial and lateral, *n* (%)	5 (13.51%)
Pattern of tears	
Radial	11 (29.72%)
Horizontal	10 (27.02%)
Bucket-handle	16 (43.24%)

Age and BMI are presented as mean ± standard deviation, whereas symptom variables are reported as median (IQR).

### Gait phase analysis

**Figure 2:**
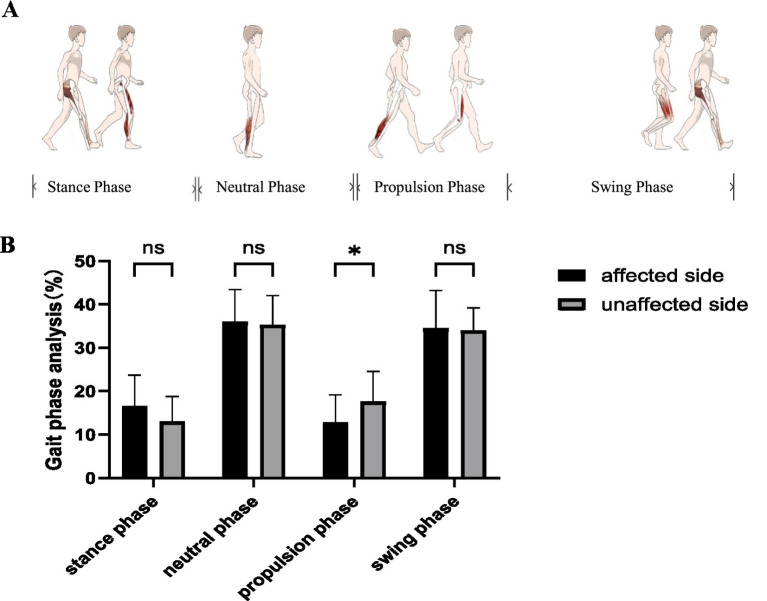
Gait phase analysis. **(A)** Gait phase analysis involves the stance phase, neutral phase, propulsion phase, and swing phase. **(B)** The results of the gait phase analysis indicated that the affected side displayed a shorter phase duration during the propulsion phase compared to the unaffected side. ns, no significant differences. *p < 0.05.

The results of the gait phase analysis indicated that the affected side displayed a shorter phase duration during the propulsion phase compared to the unaffected side [11.37 (8.39 ~ 18.91) vs. 119.48 (9.53 ~ 23.02), *p* = 0.008]. However, no significant differences were observed between the affected and unaffected sides during the stance phase, neutral phase, and swing phase (*p* > 0.05) (Table 2 and Figure 2).

**Table 2:** Measurement results of gait phase analysis parameters [% (medians/IQRs)].

Parameter	Affected side (*n* = 37)	Unaffected side (*n* = 37)	*Z*-value	*p*-value	Cohen’s *d*/*r*
Stance phase (%)	18.69 (10.09 ~ 22.63)	10.81 (8.98 ~ 17.19)	−1.810	0.07	0.308
Neutral phase (%)	34.34 (33.01 ~ 37.35)	34.17 (31.37 ~ 35.39)	−1.184	0.236	0.114
Propulsion phase (%)	11.37 (8.39 ~ 18.91)	19.48 (9.53 ~ 23.02)	−2.633	0.008	−0.382
Swing phase (%)	34.26 (31.69 ~ 35.17)	34.53 (32.11 ~ 36.49)	−0.626	0.541	0.062

### General parameters of gait

**Figure 3:**
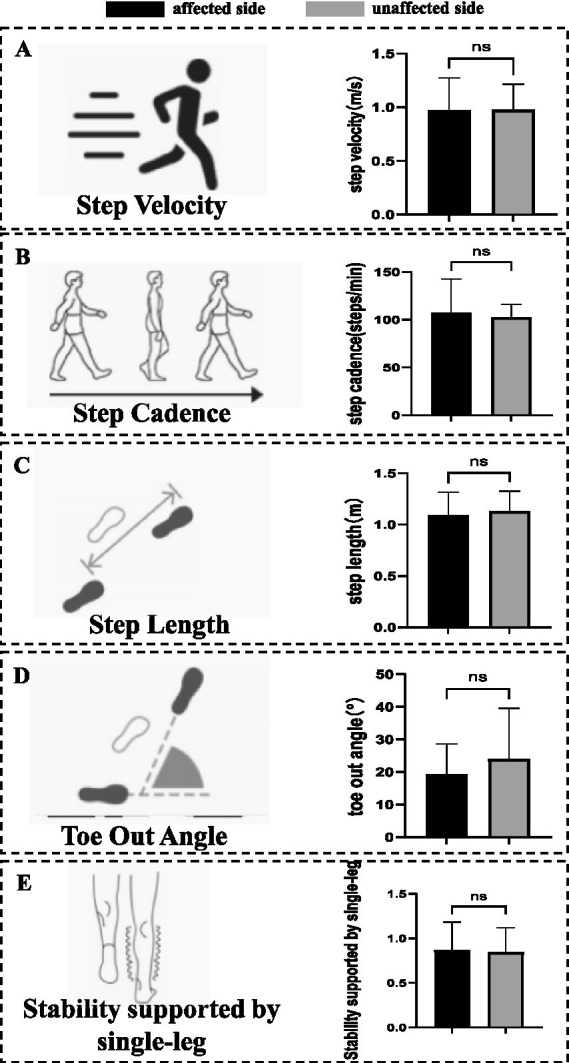
General parameters of gait. **(A)** Parameters of step velocity. No significant differences were observed in step velocity. **(B)** Parameters of step cadence. No significant differences were observed in step cadence. **(C)** Parameters of step length. No significant differences were observed in step length. **(D)** Parameters of toe-out angle. No significant differences were observed in toe-out angle. **(E)** Parameters of stability support during single-leg stance. No significant differences were observed in stability support during single-leg stance. ns, no significant differences.

The results of the general parameters of gait indicated that no significant differences were observed in step velocity, step cadence, step length, toe-out angle, and stability support during single-leg stance between the affected and unaffected sides (Table 3, Figure 3).

**Table 3:** Measurement results of general parameters of gait analysis parameters [( $\bar{x}\pm s$ ) (medians/IQRs)].

Parameter	Affected side (*n* = 37)	Unaffected side (*n* = 37)	*Z*/*t*-value	*p*-value	Cohen’s *d*/*r*
Step velocity (m/s)	0.97 (0.84 ~ 1.14)	0.96 (0.85 ~ 1.11)	−1.715	0.086	−0.001
Step cadence (step/min)	105.57 (96.95 ~ 112.61)	105.73 (97.11 ~ 112.36)	−0.830	0.407	−0.001
Step length (m)	1.09 (0.98 ~ 1.23)	1.11 (1.03 ~ 1.24)	−1.730	0.084	0.149
Toe out angle (°)	17.47 (11.62 ~ 24.5)	18.04 (11.32 ~ 31.99)	−1.667	0.096	0.148
Stability supported by single-leg	0.87 ± 0.31	0.84 ± 0.28	*t* = 0.703	0.486	−0.233

### Changes in the sagittal plane foot to ground angle

**Figure 4:**
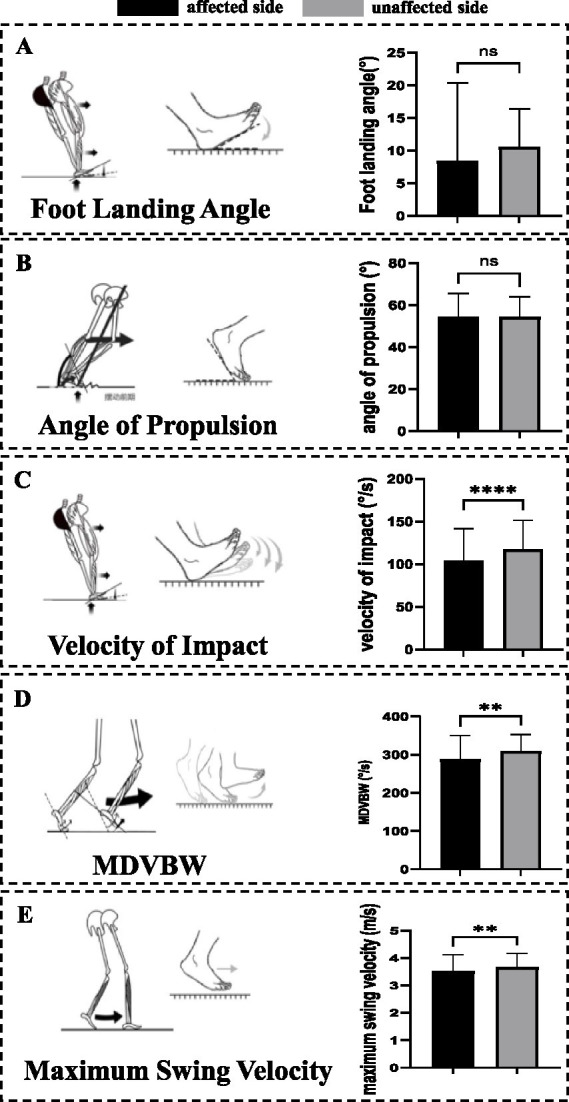
Changes in the sagittal plane foot to ground angle. **(A)** Parameters of foot landing angle. No significant differences were observed in foot landing angle. **(B)** Parameters of angle of propulsion. No significant differences were observed in angle of propulsion. **(C)** Parameters of velocity of impact. The velocity of impact on the affected side was significantly lower than that on the unaffected side. **(D)** Parameters of MDVBW. The MDVBW on the affected side was significantly lower than those on the unaffected side. **(E)** Parameters of maximum swing velocity. The maximum swing velocity on the affected side was significantly lower than those on the unaffected side. MDSBW, the maximum dorsiflexion velocity at the beginning of the swing; ns, no significant differences; **p < 0.01. ****p < 0.001.

Analysis of sagittal plane angles between the plantar surface and the ground revealed that the velocity of impact (104.27 ± 34.48 vs. 117.49 ± 33.99, *p* < 0.001), MDVBW [298.67 (258.32 ~ 321.75) vs. 301.55 (284.64 ~ 328.81), *p* = 0.010], and maximum swing velocity [3.64 (3.24 ~ 3.84) vs. 3.66 (3.37 ~ 3.99), *p* = 0.006] on the affected side were all significantly lower than those on the unaffected side. In contrast, the foot landing angle and angle of propulsion did not exhibit significant differences between the affected and unaffected sides (Table 4, Figure 4).

**Table 4:** Measurement results of sagittal plane foot-to-ground angle analysis parameters [( $\bar{x}\pm s$ ) (medians/IQRs)].

Parameter	Affected side (*n* = 37)	Unaffected side (*n* = 37)	*Z*/*t*-value	*p*-value	Cohen’s *d*/*r*
Foot landing angle (°)	11.22 (4.38 ~ 14.19)	11.51 (6.12 ~ 14.61)	−0.566	0.925	−0.161
Angle of propulsion (°)	54.85 (50.36 ~ 62.06)	54.98 (50.94 ~ 59.95)	−0.094	0.925	−0.018
Velocity of impact (°/s)	104.27 ± 34.48	117.49 ± 33.99	*t* = −4.211	<0.001	−0.674
MDVBW (°/s)	298.67 (258.32 ~ 321.75)	301.55 (284.64 ~ 328.81)	−2.565	0.010	−0.989
Maximum swing velocity (m/s)	3.64 (3.24 ~ 3.84)	3.66 (3.37 ~ 3.99)	−2.761	0.006	−0.454

MDSBW, the maximum dorsiflexion velocity at the beginning of the swing.

### Changes in the coronal plane foot to ground angle

**Figure 5:**
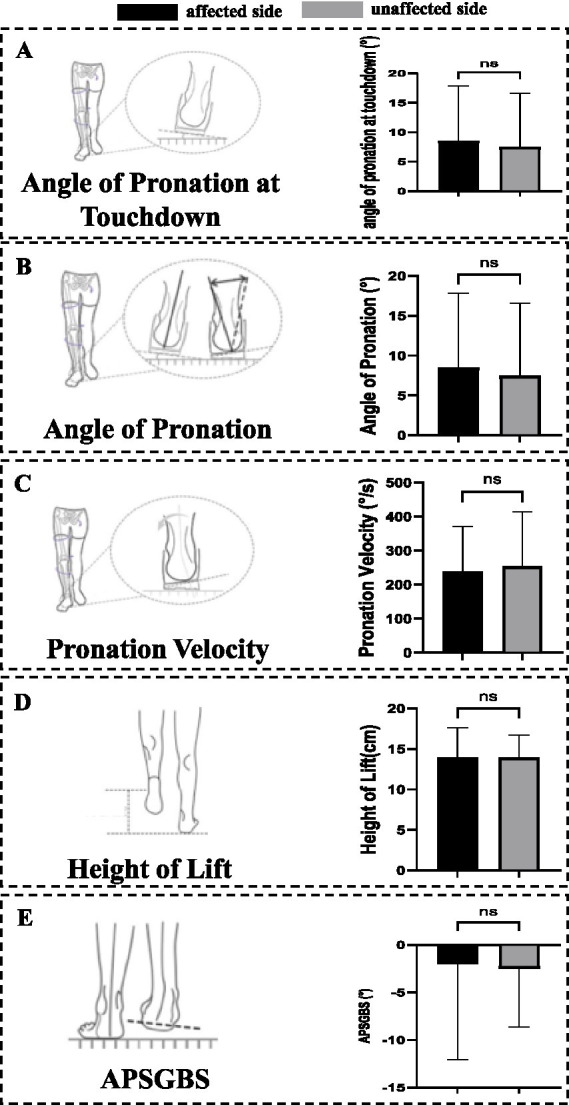
Changes in the coronal plane foot to ground angle. **(A)** Parameters of angle of pronation at touchdown. No significant differences were observed in angle of pronation at touchdown. **(B)** Parameters of angle of pronation. No significant differences were observed in angle of pronation. **(C)** Parameters of pronation velocity. No significant differences were observed in pronation velocity. **(D)** Parameters of height of lift. No significant differences were observed in height of lift. **(E)** Parameters of APSGBS. No significant differences were observed in APSGBS. APSGBS, The angle between the plantar surface of the foot and the ground at the beginning of the swing; ns, no significant differences.

There were no significant differences in the angle of pronation at touchdown, angle of pronation, pronation velocity, height of lift, and APSGBS between the affected and unaffected sides (Table 5 and Figure 5).

**Table 5:** Measurement results of coronal plane foot-to-ground angle analysis parameters (medians/IQRs).

Parameter	Affected side (*n* = 37)	Unaffected side (*n* = 37)	*Z*-value	*p*-value	Cohen’s *d*/*r*
Angle of pronation at touchdown (°)	10.94 (5.95 ~ 14.63)	9.02 (5.04 ~ 13.89)	−1.561	0.118	0.219
Angle of pronation (°)	8.17 (4.74 ~ 11.05)	7.67 (4.32 ~ 11.01)	−0.611	0.541	0.009
Pronation velocity (°)	227.2 (139.58 ~ 343.3)	221.24 (160.46 ~ 328.97)	−1.260	0.208	−0.207
Height of lift (cm)	14.23 (12.12 ~ 16.06)	14.12 (12.41 ~ 15.96)	−1.192	0.233	0.005
APSGBS (°)	0.14 (−5.12 ~ 3.37)	−2.15 (−6.22 ~ 0.89)	−1.546	0.122	−0.065

APSGBS, The angle between the plantar surface of the foot and the ground at the beginning of the swing.

### Measurement results of foot long axis and forward direction angle

**Figure 6:**
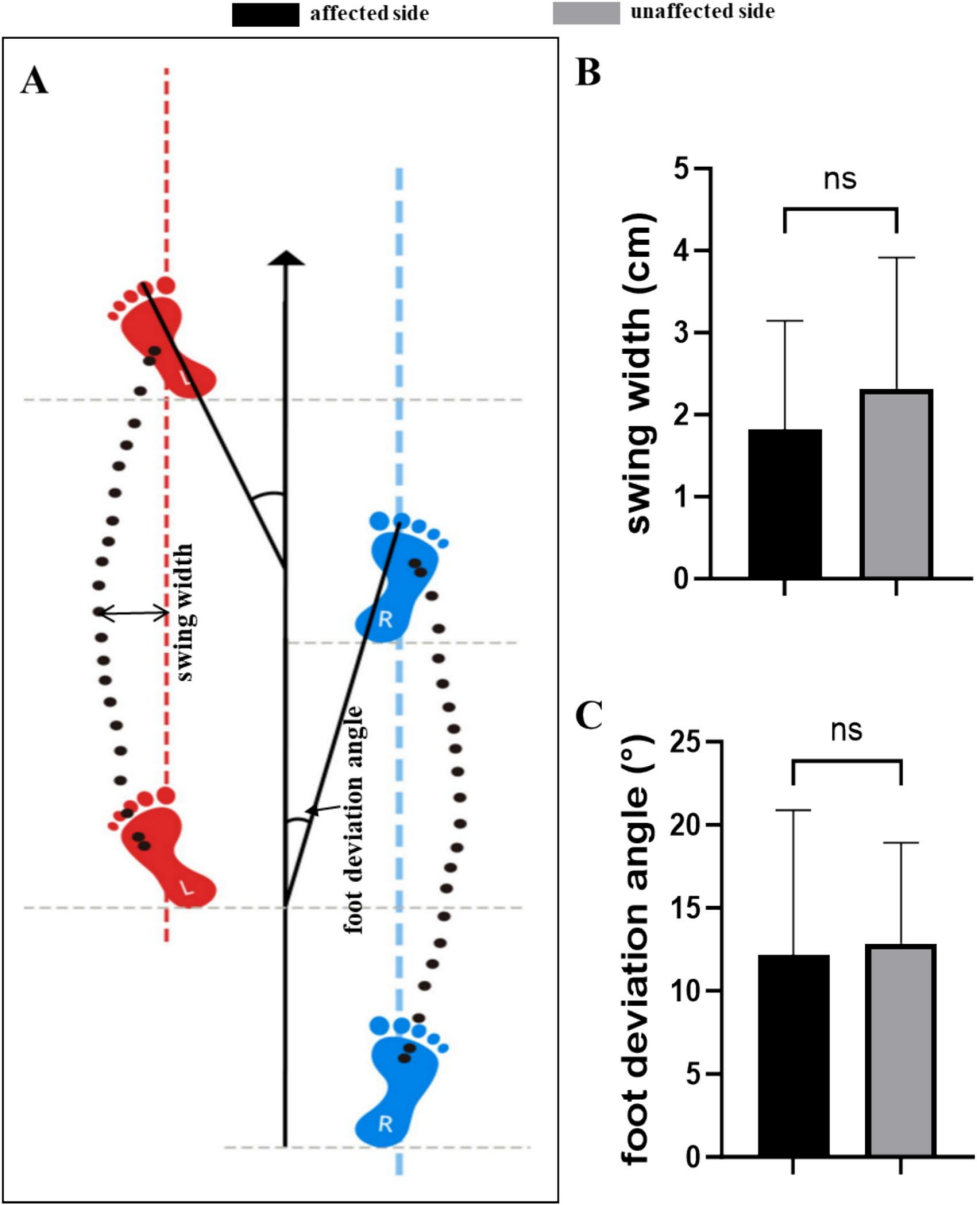
Measurement results of foot long axis and forward direction angle. **(A)** Parameters of swing width and foot deviation angle. **(B)** There was no significant difference in swing width between affected side and unaffected side. **(C)** There was no significant difference in foot deviation angle between affected side and unaffected side. ns, no significant differences.

There was also no significant difference in swing width and foot deviation angle between affected side and unaffected side (Table 6 and Figure 6).

**Table 6:** Measurement results of foot long axis and forward direction angle.

Parameter	Affected side (*n* = 37)	Unaffected side (*n* = 37)	*Z*-value	*p*-value	Cohen’s *d*/*r*
Swing width (cm)	1.52 (0.81 ~ 2.39)	1.86 (1.22 ~ 2.73)	−1.426	0.154	−0.264
Foot deviation angle (°)	10.97 (5.21 ~ 16.39)	12.85 (8.63 ~ 16.13)	−1.018	0.309	−0.081

## Discussion

This study utilized the Dynamic Gait & Posture Analysis System to measure and evaluate the gait movement feature parameters of patients with meniscus injuries, thereby obtaining the gait data of the participants. A comparative analysis was conducted between the affected and healthy sides of the patients. The results indicate that the affected side demonstrated a reduced phase during the propulsion phase when compared to the unaffected side. Additionally, analysis of the sagittal plane angles between the plantar surface and the ground revealed that the impact velocity, MDVBW, and maximum swing velocity on the affected side were all lower than those recorded on the unaffected side.

A torn meniscus in the knee is typically caused by an external force impact, rotational shearing force, or degeneration. Additionally, prolonged excessive use of the knee joint may also lead to meniscal injury. The incidence of meniscus tears increases with age. Generally, the pattern of injury varies significantly, being associated with traumatic events in younger patients and degenerative changes in adults. If not treated promptly, a meniscus tear can result in serious damage to the knee cartilage. Arthroscopic knee surgery for meniscus disease is among the most common surgical procedures. Damage to the knee cartilage may lead to early-onset osteoarthritis, impair joint function, and diminish quality of life (6). Notably, complex or multidirectional meniscus tears are linked to a higher incidence and severity of cartilage degeneration compared to other types of meniscus tears (7). Furthermore, it has been reported that moderate cartilage damage is associated with poor functional outcomes, while grade IV damage typically necessitates subsequent surgical intervention (8).

Gait analysis is a non-invasive motion capture technique commonly employed to characterize the gait patterns of specific populations and to provide insights into the functional status of the knee joint. Objective gait functional analysis can enhance the assessment of knee joint function and clinical outcomes following various treatment interventions. When compared to the self-selected walking speed of healthy individuals, individuals with semilunar and discoid lateral meniscus injuries exhibited slower walking speeds and shorter stride lengths. However, no significant differences were noted in the step frequency among the groups. The research findings align with those of other scholars who have observed significant differences in self-selected walking speed prior to surgery (9, 10). Gait analysis is frequently employed to assess lower limb diseases, encompassing both slow- and acute-onset knee conditions, which can act as an early warning system. The objectives of gait analysis can be categorized into three distinct areas: describing the gait patterns of specific populations, classifying and categorizing the extent of functional impairment within these populations, and evaluating the clinical effects of various interventions.

The gait analysis system presented in this research is founded on professional-grade algorithms that can dynamically collect fundamental data regarding the walker’s foot and display this movement data in real time. It is highly portable, enabling the collection of dynamic foot data without spatial limitations. Utilizing principles of biomechanics and big data, the system generates a data analysis model through a cloud-based algorithm, providing corresponding gait features based on the status of specific muscle groups.

In the context of chronic knee conditions, the most thoroughly investigated aspect of gait analysis is knee osteoarthritis. Numerous studies have compared gait patterns between patients with knee osteoarthritis and healthy controls (11), as well as evaluated the degree of gait abnormalities across varying severities of knee osteoarthritis (12, 13). Additionally, functional classification systems based on gait features have been proposed (14, 15), and the effects of various interventions have been evaluated (16–18). Another knee condition that has been examined is the degenerative meniscal tear. Studies have compared gait deviations between individuals with degenerative meniscal tear and healthy controls (19, 20), evaluated the impacts of diverse interventions (21), and questioned the necessity of surgical intervention within this population (22, 23). *In vitro* analysis models have demonstrated that the removal of the meniscus can lead to an increase in contact stress due to its limited shock absorption and load distribution functions (10, 24). Although these studies suggest a heightened level of knee loading following meniscus removal, further investigation is required to fully understand the implications.

According to reports (25), knee osteoarthritis can impair gait function. Additionally, the spatial and temporal features of gait parameters deteriorate, including reduced walking speed, shorter stride length, longer stride time, prolonged support phase time, and increased double support time. Recent studies have indicated that knee osteoarthritis can alter gait variability, offering crucial insights into the rhythm of the gait cycle. Both aging and various pathological conditions can modify neuromusculoskeletal behavior and disrupt the rhythmicity of the gait cycle. The greater the history of falls among community-dwelling elderly individuals, the higher their gait variability (26). A study compared pre- and post-operative gait conditions in patients who underwent partial meniscectomy. At 4 weeks postoperatively, significant reductions were observed in stride length, swing phase duration, rhythm, and speed during gait analysis within the patient group. Additionally, the standing phase duration and total double support were higher for these patients, who exhibited smaller steps and slower walking speeds. At 12 weeks postoperatively, differences persisted in standing phase duration, swing phase duration, and total double support data; however, disparities in other parameters had disappeared. The initial measurement did not reveal any differences in stride width data between the two groups. However, a significant discrepancy was observed in stride width data for the patient group during the second measurement (19). However, they did not examine kinematic variables from adjacent joints—such as the propulsion phase, impact velocity, MDVBW, and maximum swing velocity—which can shed light on whole-limb strategies after meniscal injury and help inform clinical decision-making.

In our study, the affected limb spent a smaller proportion of the gait cycle in the push-off (propulsion) phase than the unaffected limb. This pattern suggests that meniscal pain may lead patients to dampen plantarflexor and/or knee extensor involvement during gait to avoid excessive knee flexion–extension. Larger flexion–extension excursions are typically coupled with greater internal tibial rotation, which can increase compressive and shear loading on the injured meniscus (27, 28)—a classic pain-avoidance strategy.

Sagittal foot-to-ground angle analysis further showed that MDVBW (primarily the tibialis anterior) was lower on the affected side at early swing, indicating a pain-related suppression of rapid tibialis-anterior activation (29) to limit anterior tibial translation from overly fast dorsiflexion. In addition, the reduction in maximum swing velocity implies a slower knee flexion velocity, consistent with a compensatory approach aimed at reducing meniscal compressive load. Such neuromuscular adaptations may be acutely beneficial for protecting the injured tissue. However, if this abnormal movement pattern persists, it may disrupt load sharing along the knee–ankle kinetic chain, provoke proximal compensations, and over time weaken push-off capacity of the calf on the affected side (30). Meanwhile, the contralateral limb may assume an excessive share of propulsion, increasing the risk of chronic overload at the contralateral ankle (31).

We found no significant differences in frontal-plane parameters. This likely reflects the fact that meniscal pathology primarily alters flexion–extension mechanics in the sagittal plane. The meniscus plays a central role in shock absorption and load distribution during knee flexion and extension (32), whereas varus–valgus stability in the frontal plane depends more on the collateral ligaments and proximal (hip/trunk) control. During level walking, patients can rely on hip abductors and trunk strategies to maintain frontal-plane balance, which may mask between-limb differences in frontal metrics (33). Moreover, frontal-plane excursions are smaller than those in the sagittal plane, creating a “floor effect” that makes subtle compensations harder to detect with gross gait parameters.

In other words, pain-avoidance control after meniscal injury tends to present as “lower flexion–extension velocity” and “reduced push-off force” during propulsion and swing, rather than as changes in side-to-side (frontal-plane) control. This also suggests that rehabilitation should prioritize rebuilding the sagittal-plane kinetic chain-particularly coordinated knee–ankle flexion–extension training.

This study has several limitations. It used a cross-sectional design with a relatively small sample, which may restrict the generalizability of the findings. Furthermore, the analysis did not distinguish between medial and lateral meniscus injuries, potentially masking location-specific gait patterns. A more critical limitation is the absence of a healthy control group. Although comparing the affected and contralateral limb is common, the unaffected side may already show compensatory adaptations. As a result, the true extent of impairment may be underestimated. In addition, due to the sample size limitation, no further analysis was conducted on this subgroup that did not target the injury time. Future studies should include healthy participants and perform subgroup analyses based on lesion location.

## Conclusion

Following a meniscus lesion, we observed reductions in propulsion-phase proportion, impact velocity, MDVBW, and maximum swing velocity in the affected limb. These alterations reflect limb-specific, pain-avoidance adaptations along the knee–ankle sagittal kinetic chain. Taken together, these side-to-side asymmetries may serve as functional markers that complement imaging, inform hypothesis generation, and help tailor rehabilitation targets; however, their prognostic or diagnostic utility requires validation in prospective, controlled cohorts.

## Data Availability

The raw data supporting the conclusions of this article will be made available by the authors, without undue reservation.
